# Fatigue Life of Austenitic Steel 304 Bolts Strengthened by Surface Treatment with Graphene Oxide Layer and Surface Shot Peening

**DOI:** 10.3390/ma14216674

**Published:** 2021-11-05

**Authors:** Barbara Nasiłowska, Zdzisław Bogdanowicz, Sylwester Kłysz, Marta Baran, Janusz Lisiecki, Grzegorz Mońka, Bartosz Bartosewicz, Zenon Komorek, Aneta Bombalska, Zygmunt Mierczyk

**Affiliations:** 1 Institute of Optoelectronics, Military University of Technology, gen. S. Kaliskiego 2, 00-908 Warsaw, Poland; bartosz.bartosewicz@wat.edu.pl (B.B.); aneta.bombalska@wat.edu.pl (A.B.); zygmunt.mierczyk@wat.edu.pl (Z.M.); 2 Faculty of Mechanical Engineering, Military University of Technology, gen. S. Kaliskiego 2, 00-908 Warsaw, Poland; zdzislaw.bogdanowicz@wat.edu.pl; 3 Air Force Institute of Technology, Księcia Bolesława 6, 01-494 Warsaw, Poland; sylwester.klysz@itwl.edu.pl (S.K.); marta.baran@itwl.edu.pl (M.B.); Janusz.Lisiecki@itwl.edu.pl (J.L.); 4 Faculty of Technical Science, University of Warmia and Mazury, Oczapowskiego 11, 10-719 Olsztyn, Poland; 5 Łukasiewicz Research Network—Institute of Precision Mechanics, Duchnicka 3, 01-796 Warsaw, Poland; monka@imp.edu.pl; 6 Faculty of Advanced Technology and Chemistry, Military University of Technology, gen. S. Kaliskiego 2, 00-908 Warsaw, Poland; zenon.komorek@wat.edu.pl

**Keywords:** graphene oxide, shot peening, screw, austenitic steel 304, fatigue life

## Abstract

This paper presents the results of investigations of the effect of graphene oxide and surface shot peening on the mechanical properties and fatigue life of bolts made of austenitic 304 steel. An innovative method for the uniform deposition of graphene oxide on screws is presented. The process involved activating the surface using plasma and then performing graphene oxide deposition using centrifugal force and vacuum drying. The screw specimens prepared in this way were subjected to a surface peening process. Comparative studies have shown that the combination of graphene oxide deposition and shot peening processes results in an increase in fatigue life of approximately 42 ÷ 275% (depending on the stress amplitude level) compared to the as-delivered samples. The results presented are promising and may provide a basis for further research on the application of graphene and its derivatives to increase fatigue life and improve the mechanical properties of machine components.

## 1. Introduction

Modification of the surface layer of machine components can have a significant effect on improving their mechanical properties [1,2,3,4,5,6,7]. One important factor in the initiation and development of fatigue cracks concerns structural and geometric notches, which cause local accumulation of stresses favoring the weakening of the structure [7]. To extend the service life of machine elements, it is advisable to strengthen the surface layer by performing, for example, an appropriate treatment that introduces a favorable state of compressive stresses in the surface layer [1,2,4]. Such reinforcement can be done, for example, by shot peening of the surface [1,2,4]. Soyama [4,7,8,9,10,11,12,13], Takakuwa [12,13], and Nakonieczny [14,15] devoted numerous scientific publications to the effect of the shot peening process on the structural, mechanical, and fatigue properties of structural materials.

They showed that the surface crushing created by surface peening leads to compressive stresses in the subsurface layers affecting crack development. In non-surface-strengthened specimens, crack initiation usually occurs at the bottom of structural or geometric notches, whereas the introduction of a state of subsurface compressive stresses can shift the crack initiation site to the point of maximum material stress below the surface [5,15] and, most importantly, increase fatigue life.

The current technological challenge is to combine the strengthening process occurring during surface shot peening with the introduction into the surface layer of a material having different properties than the native material to improve its mechanical and fatigue properties.

The combination of graphene and its derivatives with metal can significantly improve the mechanical properties [6,16,17]. Although research on graphene and its derivatives is expanding [18,19,20,21,22,23,24], this effect has rarely been studied due to the difficulty in obtaining a uniform dispersion. The tendency for uneven distribution and agglomeration of graphene flakes is important in components that have varying shapes and sizes.

The literature contains information on composites in which graphene has been bonded to the base material directly [16,25] or via carriers such as titanium [26], aluminum [27,28,29], or copper [30]. Methods of the fabrication of graphene nanosheet (GNS) composites have also been described [29]. During conventional production methods, such as powder metallurgy or rolling, graphene and its derivatives were embedded in the matrix of the composite material. However, the effect of graphene oxide deposition on the mechanical and fatigue properties of such structures has not been presented and sufficiently studied so far, which results in a negligible number of scientific articles devoted to this issue.

The aim of this research was to analyze the effect of graphene oxide deposition and surface shot peening on mechanical and fatigue properties. The tests were conducted on a structural element, such as a screw, which had a surface geometry that had numerous geometric notches making the uniform deposition of graphene oxide more complicated.

## 2. Materials and Methods

### 2.1. Materials

#### 2.1.1. Object of Research

**Figure 1:**
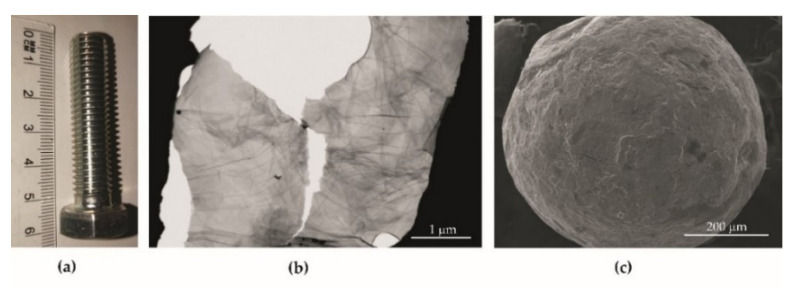
Object of research: optical image of the screw as supplied (**a**); STEM flakes of graphene oxide (GO) (**b**); SEM image of shot peening (**c**).

The tests were performed on M12x50 bolts (Figure 1a) made of A2 stainless steel (according to the following designations: EN 1.4301, PN 0H18N9, AISI 304, X5CrNi18-10, GOST 08Ch18N10, SS 2332, and CSN 17240), with a chemical composition expressed in % of Ni 8.0–10.5; Cr 17.5–19.5; Mn < 2.0; C < 0.07; Si < 1.0; *p* < 0.045; S < 0.015; *n* < 0.11; Fe = bal. For static strength and fatigue life testing, the bolt head was removed.

#### 2.1.2. Graphene Oxide

Graphene oxide (GO) flakes dispersed in water (Figure 1b) were obtained from flake graphite by a modified Hummers method (Department of Chemical Synthesis and Flake Graphene, Łukasiewicz Research Network, Institute of Electronic Materials Technology, Warsaw, Poland). Flake graphite (Asbury Carbons, St., Asbury, NJ, USA) was added to a reactor containing concentrated sulfuric acid (H_2_SO_4_, Chempur, Poland) and phosphoric acid (H_3_PO_4_, Chempur, Poland). Next, potassium permanganate (KMnO_4_, Chempur, Poland) was slowly added to this mixture. The oxidation process was performed for a few hours and stopped by the addition of deionized water and hydrogen peroxide (30% H_2_O_2_, Chempur, Poland). The graphite oxide water suspension obtained was left for sedimentation. Then, the purification and exfoliation processes were performed. The concentration of graphene oxide water dispersion was 10 g/L [31,32]. The flake size was 3–10 µm and the graphene oxide was purchased from the Department of Chemical Synthesis and Flake Graphene, Łukasiewicz Research Network, Institute of Electronic Materials Technology, Warsaw, Poland.

#### 2.1.3. Shot Peening

Shot peening media (i.e., diameter and material) were selected so that during peening, the balls could move freely to reinforce the bottom of the geometric notch without carrying shot material other than the bolt’s parent material. The transfer of material other than the bolt’s parent material could result in the initiation of corrosion and, therefore, geometric and structural weakening of the structural component during its service life. Thus, an austenitic steel shot was used which had an elemental composition similar to the bolt’s parent material which was C~0.17%; Cr~18%; Ni~10%; Fe = bal. (Figure 1c).

### 2.2. Methodology of Sample Preparation

**Figure 2:**
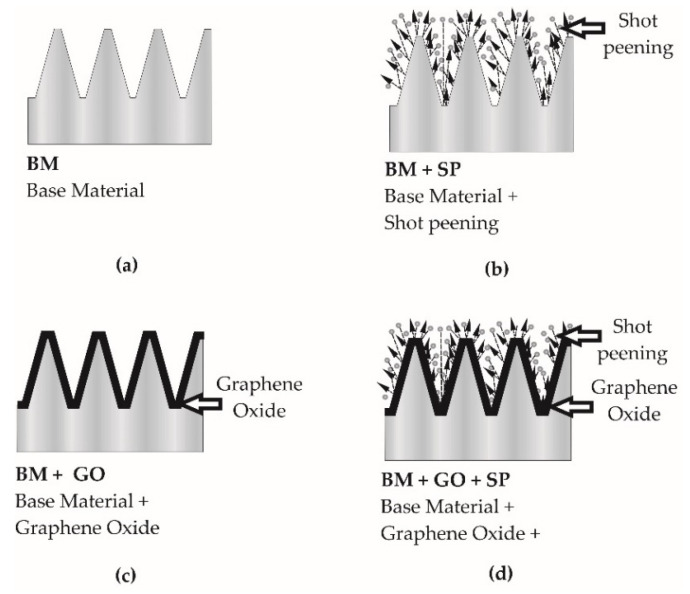
Scheme of sample preparation: BM (**a**), BM+SP (**b**), BM+GO (**c**), BM+GO+SP (**d**).

Tests on the structural, mechanical, and fatigue properties were carried out for bolts made of austenitic 304 steel according to the following designations:

- BM—base material (untreated bolts, as supplied) (Section 2.1.1) (Figure 2a);
- BM+SP—base material (Section 2.1.1) and shot peening (Section 2.1.3) (Figure 2b);
- BM+GO—base material (Section 2.1.1) and with deposited graphene oxide layer (Section 2.2.2) (Figure 2c);
- BM+GO+SP—base material (Section 2.1.1) and with a deposited graphene oxide layer (Section 2.2.2) and shot peened (Section 2.1.3) (Figure 2d).

#### 2.2.1. Deposition of Graphene Oxide on the Screw Surface

**Figure 3:**
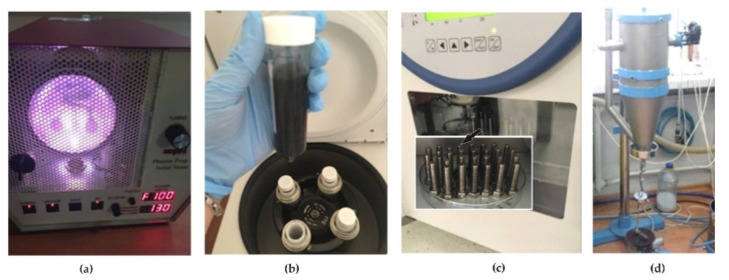
Workstation for plasma cleaning and surface activation (**a**); deposition of graphene oxide on screws (**b**); vacuum drying of screws (**c**); surface peening (**d**).

The graphene oxide deposition process was performed for a part of the bolts in the delivery state (BM+GO) and bolts in the delivery state intended for subsequent shot peening (BM+GO+SP). The process consisted of plasma cleaning and surface activation (Figure 3a), graphene oxide deposition (Figure 3b), and vacuum drying (BM+GO) (Figure 3c). Some of the samples prepared in this way were additionally subjected to the surface peening process (BM+GO+SP) Section 2.2.2 (Figure 3d).

In the first step, the surface cleaning and activation process was optimized by plasma exposure (100 W for 30 min) in an N_2_ atmosphere (Figure 3a).

The cleaning and activation of the sample’s surface by RF plasma is crucial in the deposition of graphene oxide, because it promotes the hydrophilicity of the steel. As a result, the graphene oxide aqueous suspension (described in Section 2.1.2) spreads well over the steel surface facilitating uniform distribution and adherence of graphene oxide flakes [33,34].

**Figure 4:**
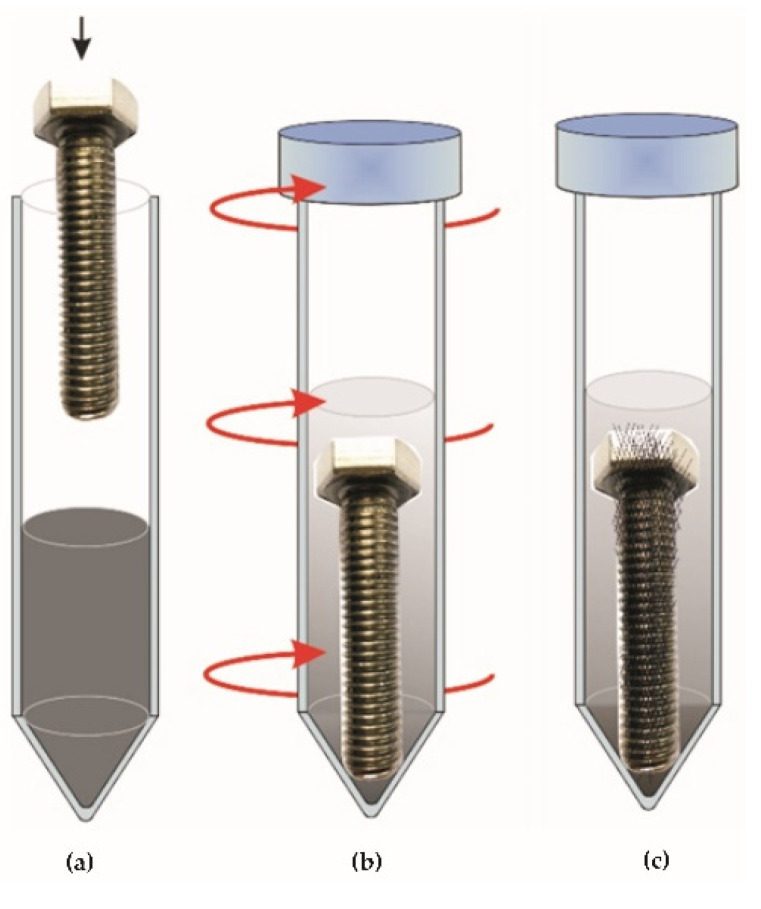
The graphene oxide deposition process on screws: after plasma cleaning, immersion in GO suspension (**a**); exposure to centrifugal force in a centrifuge (**b**); repeated centrifugation in a clean Falcone tube (**c**).

Immediately after removing the samples from the chamber in which plasma acted on them, they were immersed in a dispersed aqueous suspension of graphene oxide with a concentration of 10 g/L located in the Falcone tubes (Figure 3b and Figure 4a).

The process of plasma interaction positively influences the surface cleaning and adhesive incorporation of graphene oxide particles into the surface layer. Next, the Falcone tubes with the samples were placed in the rotor of the centrifuge and subjected to centrifugal force (3 rpm at 40 °C) (Figure 4b). To separate the GO excess, the samples were removed from the Falcone tubes containing the suspension and placed in clean Falcone tubes without the suspension and then subjected to a second centrifugation process under the same parameters (Figure 4c). The process of centrifugation of samples in Falcone tubes containing no graphene oxide suspension was performed twice. In the last step, the samples were placed in a vacuum dryer for 24 h at a temperature of 50 degrees C (Figure 3c). Then, after the graphene oxide deposition process was performed in this way, the surface peening process was performed on BM+GO+SP samples (Figure 3d).

#### 2.2.2. Shot Peening Process

The surface peening process was carried out for the bolt parts in the delivery state (BM+SP) and with a deposited graphene oxide layer (BM+GO+SP). A special workstation for shot peening type PEEN—IMP (proprietary peening station in Institute of Precision Mechanics) (patent PL 204718) was used, which enabled the smooth regulation of the energy of the shot impact on a strengthened surface. The shot pellet granulation and process parameters were selected experimentally so that the shot peened surface coverage was 100%. During the shot peening process, the specimen rotated in a rotary motion, and the shot hit perpendicularly to the surface of the specimen. The following peening parameters were selected: stainless-steel shot with diameters of 0.4 and 0.6 mm, air pressure of *p* = 5 bar, and an exposure time of 15 min. The diameter of the shot was selected to strengthen the bottom of the thread. The intensity of peening measured on Almen-type NI plates was *f_N_* = 0.24 mm. The distance of the nozzle assembly from the shot peened surface was *l* = 420 mm.

### 2.3. Characterization by Scanning Electron Microscopy

Graphene oxide was deposited on screws investigated by scanning electron microscopy (SEM, Hillsboro, OR, USA) and scanning transmission electron microscopy (STEM, Hillsboro, OR, USA) using a Quanta 250 FEG SEM, FEI, Hillsboro, OR, USA. SEM images were acquired using a backscattered detector (ETD-BSE, FEI, Hillsboro, OR, USA) with an accelerating voltage of 2–5 kV for GO and 5–10 kV. 

### 2.4. Surface Morphology Raman 

The Raman spectra were acquired using a Renishaw InVia Raman microscope equipped with an Andor EMCCD detector (Renishaw plc., Wotton-under-Edge, UK). The Raman signal was collected using laser radiation with a wavelength of 532 nm and a laser excitation power of 2.5 mW. The laser beam was directed to the sample through a 20× objective lens, and the laser spot size was approximately 5 μm in diameter.

### 2.5. Microhardness

Microhardness measurements were performed using the Vickers method according to the requirements of PN-EN ISO 6507-01:2018-05. The tests were carried out on a Shimadzu M microhardness tester with a load of 100 g (HV0.1) and a load time of 5 s. Microhardness was determined according to the analytical relation using the averaged diagonal of the resulting imprint. Prior to the basic measurements, the measuring system was checked using the 43722 HV0.1 hardness standard No. 4480. The variation in the microhardness of the materials tested was determined based on linear microhardness distributions from the surface of the tested material to a depth of 2 mm.

### 2.6. Static and Fatigue Tensile Tests

Static tensile tests and fatigue tests were performed using a servo-hydraulic testing system (322.31 test system, MTS, Eden Prairie, MN, USA) with a load cell range equal to ±250 kN and an LVDT sensor range of ±80 mm. Specimens as screws without heads were mounted in fixtures which allowed for their axial loading.

An application toolset supplied by the machine producer was used for static tensile tests—MultiPurpose TestWare Software 793 (MPT). Tests were carried out at room conditions (23 ÷ 25 °C and 40 ÷ 60% RH) with a constant speed of actuator movement, that is to say 1 mm/min (displacement rate) and with a preload of 100 N. 

Fatigue tests were designed and conducted using another producer application, 790.20 Cyclic Fatigue under force control. The control signal had a sine wave form at a frequency of *f* = 20 Hz and a load ratio of *R* = 0.1. Tests were performed on three levels of loads: maximum force values in the load cycle corresponded to 0.8, 0.7, and 0.6, respectively, of the tensile strength obtained during the tests of the base screw material. The data, which were recorded during tests, included the number of cycles and peak and valley values of force and displacement. 

## 3. Results and Discussions

### 3.1. Surface Morphology

**Figure 5:**
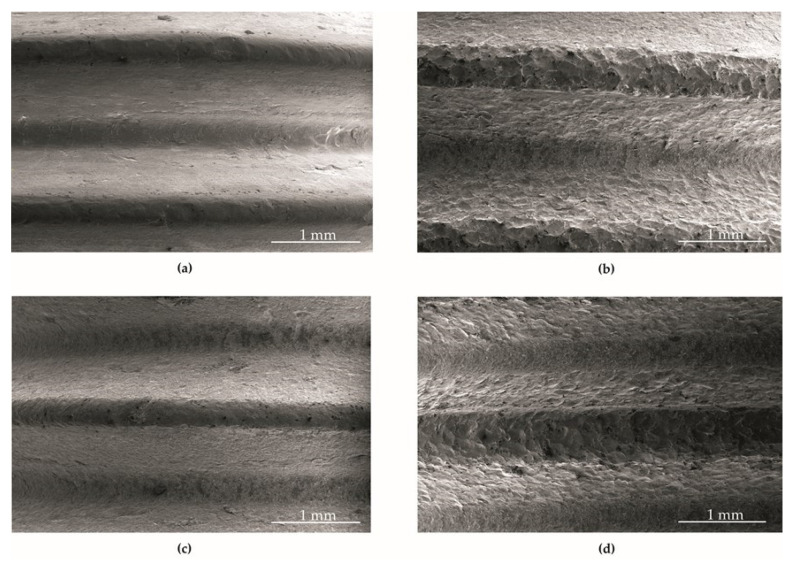
SEM image of the object of research: as delivered (BM) (**a**); shot peened (BM+SP) (**b**); with a deposited graphene oxide layer (BM+GO) (**c**); with both a deposited graphene oxide layer and shot peened (BM+GO+SP) (**d**).

Analysis of the surface morphology of the bolts made of austenitic 304 steel showed a visible development of the surface after shot peening. The surface morphologies of the screw fragments in the delivery state (BM) (Figure 5a), shot peened (BM+SP) (Figure 5b), with a deposited graphene oxide layer (BM+GO) (Figure 5c), and with a deposited graphene oxide layer and shot peened (BM+GO+SP) (Figure 5d) are presented below.

**Figure 6:**
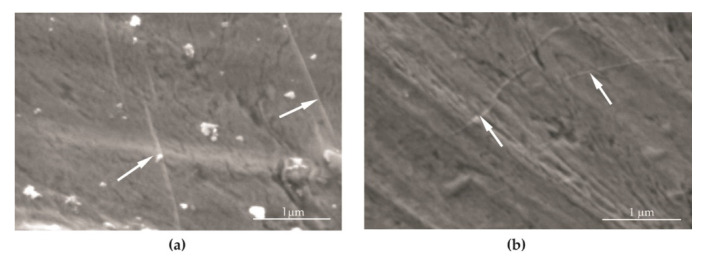
SEM image of graphene oxide in surface of the screw (**a**,**b**).

As a result of the plasma surface activation, the centrifugal force of the graphene oxide suspension on the screw, and the vacuum drying process, a uniform distribution of graphene oxide flakes was observed. This was not only on the outer surface but also in the bottom of the geometric notches, meaning the bottom of the rounded root. The deposited graphene oxide had characteristic thin folds (Figure 6a,b) located at the flake junctions and on its surface (white arrows Figure 6a,b).

**Figure 7:**
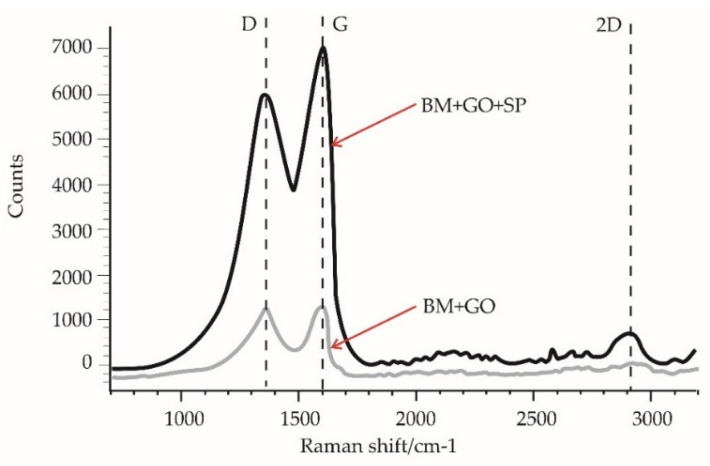
Raman spectrum of BM+GO and BM+GO+SP.

SEM microscopic analysis of the presence of graphene flakes after shot peening was difficult to interpret due to the significant surface development. Therefore, Raman spectroscopy studies of BM+GO and BM+GO+SP screw surfaces were performed The Raman spectral lines D—1360 cm^−1^, G—1600 cm^−1^, and 2D—2920 cm^−1^, typical for graphene oxide, were observed on the surface of tested BM+GO and BM+GO+SP (Figure 7) samples. The study shows that shot peening of the screw surface with a deposited GO layer (BM+GO+SP) did not completely remove the graphene oxide flakes. It is possible that by compacting the GO layer on the screw’s surface after shot peening, the voids between the graphene oxide layers are eliminated. The resulting layer mimics single GO layers, so the Raman spectra differ. Different scattering of the laser beam on the GO coating causes changes in the intensity of the D and G bands, which are not necessarily related to the actual loss of GO flakes. 

### 3.2. Microhardness 

The bolts are annealed and hardened during the manufacturing process, which makes the microhardness of the surface layer higher than that of the parent material (304 steel) not subjected to ennobling treatment.

The study shows that under the influence of the graphene oxide deposition process, the microhardness at a distance of 0.08 ÷ 0.2 mm from the front of the sample remains below 400 HV. This is due to the activation of the surface because of the high plasma temperature in the first step of graphene oxide deposition.

**Figure 8:**
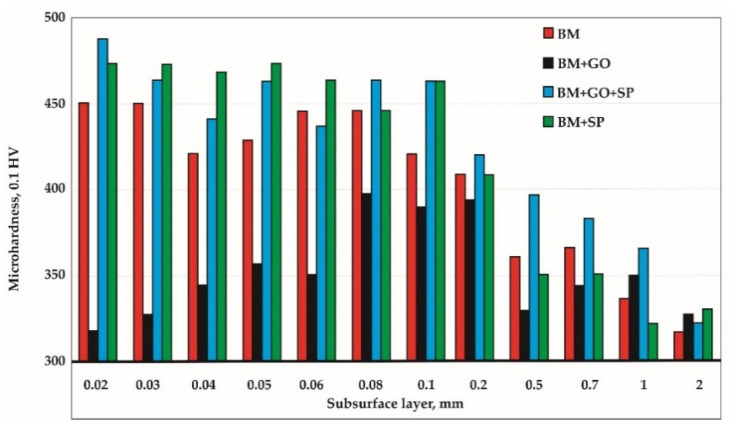
Microhardness of the surface layer of samples in delivery condition (BM), shot peened (BM+SP), with a deposited graphene oxide layer (BM+GO), and with a deposited graphene oxide layer and shot peened (BM+GO+SP).

Shot peening of the screw’s surface caused an increase in the HV microhardness of the samples (BM+SP) and those subjected to graphene oxide deposition (BM+GO+SP) (Figure 8). The change in the structure of the surface layer after the graphene oxide deposition process resulted in a greater strengthening of the surface layer of BM+GO+SP samples compared to BM+SP (Figure 8).

Samples with a deposited graphene oxide layer were subjected to plasma cleaning treatment, during which the surface layer was tempered. This resulted in lowering their microhardness to 0.06 mm, which can be clearly seen in the graph for the BM+GO samples compared to the BM material in the delivery state (Figure 8). The microhardness of the parent material of the BM bolts at 0.04 ÷ 0.06 mm was reduced due to the manufacturing process. This was probably influenced by the formation of higher microhardness in this area of the BM+SP samples compared to the BM+GO+SP samples.

The increase in the microhardness of the shot peened samples with a deposited graphene oxide layer (BM+GO+SP) in comparison to shot peened samples without a graphene oxide deposition (BM+SP) occurred at a depth of 0.08 ÷ 1 mm and was >17%. The microhardness equilibration for all samples occurred at a depth of the subsurface layer of approximately 2 mm.

### 3.3. Static and Fatigue Tensile Tests

**Figure 9:**
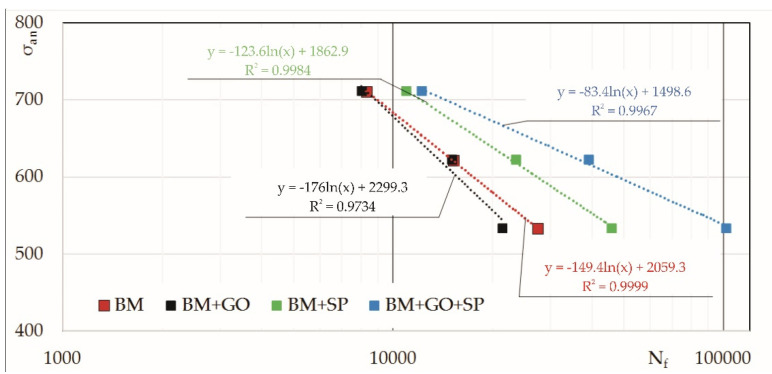
Fatigue life (N_f_) of the samples in the as-delivered state (BM), shot peened (BM+SP), with a deposited graphene oxide layer (BM+GO), and with a deposited graphene oxide layer and shot peened (BM+GO+SP), depending on the stress amplitude level, σ_an_.

The results of fatigue life tests (averaged from three tests) for each level of stress amplitude, *σ_an_*, and type of samples considered (i.e., BM, BM+SP, BM+GO, and BM+GO+SP) are presented in Table 1 and Figure 9.

Analysis of the static tensile test results showed that the scatter of the results for the different samples was within 5% of the statistical error. No significant increase in tensile strength Rm was observed for the graphene oxide deposited and shot peened (BM+GO+SP) samples compared to the other samples tested. The results of the tensile strength limit Rm were as follows: BM—885 MPa, BM+SP—878 MPa, BM+GO—887 MPa, and BM+SP+GO—893 MPa.

Figure 9 shows the fatigue lifeline equations of the Wöhler curves in the limited stress range. Investigations have shown that shot peening alone causes an increase in fatigue life (BM+SP) in comparison with the specimens not subjected to the shot peening process (BM and BM+GO). However, the highest fatigue life was observed for the samples after graphene oxide deposition and surface peening (BM+GO+SP).

The use of combined treatment of graphene oxide deposition and shot peening increased the fatigue life depending on the level of stress amplitude, σ_an_ (Figure 9). For example, at σ_an_ = 534 MPa, the graphene oxide deposited and shot peened (BM+GO+SP) samples exhibited a 122% higher fatigue life compared to the shot peened (BM+SP) samples.

Deposition of graphene oxide on the surface of bolts in the as-delivered condition (BM+GO) resulted in a reduced fatigue life compared to the untreated bolts (BM). This was caused by the partial temperability of the surface layer during plasma interaction in the graphene oxide deposition process.

### 3.4. Fracture Pattern and Microfractography of Fatigue Fractures

**Figure 10:**
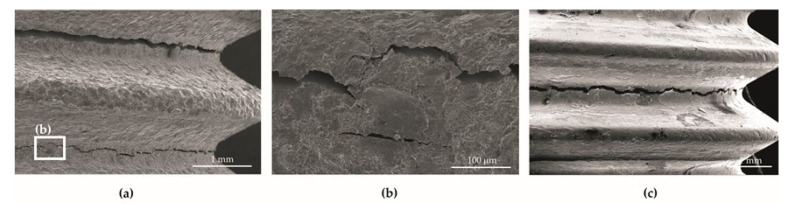
Microfractography of the thread surface after fatigue tests: samples at (BM+GO+SP) 100× (**a**); 1000× (**b**); (BM+GO) 75× (**c**).

The crack initiation in all of the samples in the delivery condition (BM) and subjected to the treatments analyzed (i.e., BM+GO, BM+SP, and BM+GO+SP) occurred at the bottom of the geometric notches, that is to say at the bottom of the rounded root thread (Figure 10a).

It was observed that the front-line fractures in the graphene oxide deposited and shot peened samples (BM+GO+SP) (Figure 10a,b) compared to the other tested samples (e.g., BM+GO) (Figure 10c) passed in two planes parallel to each other in an adjacent rounded root thread. The analysis of the microfractography of the thread surface performed with an SEM microscope showed numerous cracks also with an orientation parallel to the main crack front (Figure 10b).

**Figure 11:**
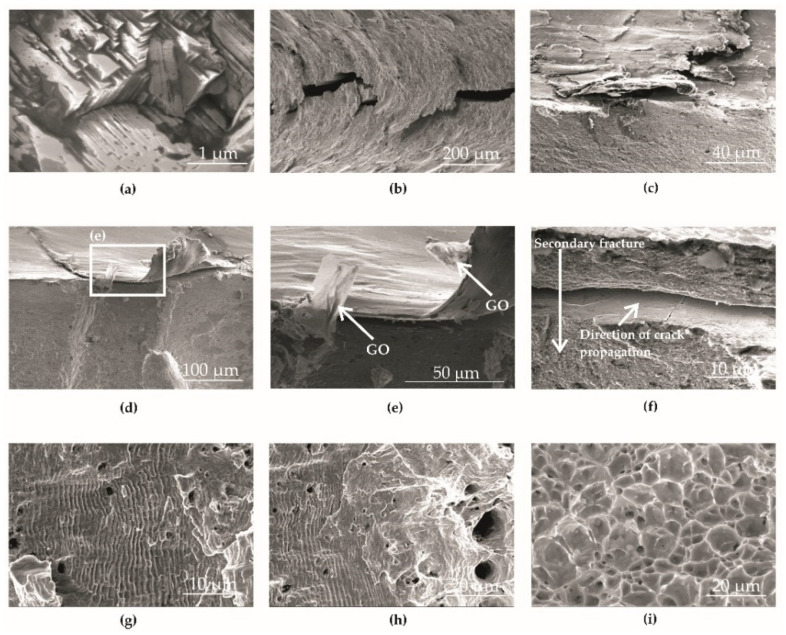
Microfractography of bolt fatigue fracture: fracture focus (**a**); thread base crack (**b**); BM+SP specimen fracture growth (**c**); BM+GO+SP specimen fracture growth (**d**–**f**); fatigue striations (**g**); transition zone (**h**); residual zone (**i**).

Observation of the fatigue fracture microfractography in the crack initiation areas showed the occurrence of brittle cracks (Figure 11a). As a result of shot peening in the surface layer, the material was strengthened, which caused an increase in the microhardness and compressive residual stresses [15,35]. The highest strengthening and tangential stresses after the shot peening process, according to the Hertz model, occurred below the surface [15]. As a result, despite the occurrence of the effects of partial decohesion of the surface layer and surface micro-roughness (Figure 11b–f), the total strengthening effect after shot peening favored an increase in the mechanical properties and fatigue life. The specimens subjected to surface strengthening exhibited a longer life span.

Examples of material decohesion and surface layer separation were observed in the bottom of the notch (Figure 11b) and on the surface of the BM+GO+SP (Figure 11c,d) and BM+SP (Figure 11e,f) samples. The graphene flake fragments are indicated by the arrow in Figure 11d.

The peened samples with deposited graphene oxide BM+GO+SP exhibited subsurface secondary cracks with the crack front perpendicular to the main crack front line (Figure 11f).

Microfractography of the fatigue fractures of all specimens revealed fatigue stripes (Figure 11g), a transition zone (Figure 11h), and a pit zone (Figure 11i). The fatigue stirpes were arranged perpendicularly to the direction of the crack front line corresponding to the position of crack tip propagation at one cycle of load change. In the transition zone separating the fatigue zone from the residual zone, plastic cracking was observed, with fatigue striations appearing (Figure 11h). The final stage of failure (pit zone) occurred because of the characteristic separation in the static rupture process [36]. The pit zone had cavities and craters where non-metallic inclusions were observed at the bottom. This included manganese sulfide (MnS) revealed by weak cohesion forces between them and austenite grains. In all samples, the residual zone was present in the middle part of the sample.

## 4. Conclusions

A comparative study of samples in the delivery condition (BM), shot peened (BM+SP), with a deposited graphene oxide layer (BM+GO), and with a deposited graphene oxide layer and shot peened (BM+GO+SP) showed that:

- The innovative process developed combining surface cleaning and activation, graphene oxide application, and vacuum drying resulted in permanent deposition of graphene oxide on the surface of screws;
- The collected Raman spectra confirmed that graphene oxide flakes were still present on the surface after the shot peening process;
- The graphene oxide deposition process combining plasma interaction and graphene oxide deposition resulted in a decrease in the microhardness of the subsurface layer of approximately 15–20% compared to the samples in the delivery state;
- For samples with a deposited graphene oxide layer and shot peened (BM+GO+SP) compared to shot peened samples without a deposited graphene oxide layer (BM+SP), an increase of more than 17% in microhardness up to a <0.2 mm depth of the subsurface layer was observed. This was the effect of graphene oxide being introduced into the surface layer during the shot peening process;
- At a depth of approximately 2 mm of the subsurface layer, the microhardness of all samples tested (i.e., BM, BM+SP, BM+GO, and BM+GO+SP) was the same;
- Samples (BM+GO+SP) had the highest fatigue life after graphene oxide deposition and shot peening;
- The use of the combined techniques of surface activation treatment, graphene oxide deposition, vacuum drying, and shot peening resulted in an increase in fatigue life by 42–275% (depending on the stress amplitude).

The results of the fatigue life tests are promising and provide a basis for further research on the effect of graphene oxide and its interaction after shot peening on increasing the fatigue life of materials in machine components.

## 5. Patents

The processing of elements presented in this work consisting of plasma cleaning and surface activation, application of graphene oxide, vacuum drying, and mechanical shot peening is called *hybrid graphene treatment* and is presented in patent application P. 438715.

## Figures and Tables

**Table 1:** Averaged fatigue life results for each level of nominal stress amplitude.

Stress Level	*σ_an_* (MPa)	BM (Number of Cycles)	BM+GO (Number of Cycles)	BM+SP (Number of Cycles)	BM+GO+SP (Number of Cycles)
0.8 Rm	711	8277	8023	10,910	12,179
0.7 Rm	622	15,179	15,106	23,524	39,184
0.6 Rm	534	27,222	21455	45,861	101,996

## Data Availability

Data are contained within the article.
